# Systematic review of the effectiveness of standalone passive countermeasures on microgravity-induced physiologic deconditioning

**DOI:** 10.1038/s41526-024-00389-1

**Published:** 2024-04-25

**Authors:** Syed Shozab Ahmed, Nandu Goswami, Adam Sirek, David Andrew Green, Andrew Winnard, Leonie Fiebig, Tobias Weber

**Affiliations:** 1 Department of Family Medicine, Postgraduate Medical Education, Queen’s University School of Medicine, Kingston, ON Canada; 2 Division of Physiology, Otto Löwi Research Center for Vascular Biology, Immunity and Inflammation, Medical University of Graz, Graz, Austria; 3 College of Medicine, Mohammed Bin Rashid University of Medicine and Health Sciences, Dubai, United Arab Emirates; 4 Integrative Health Department, Alma Mater Europaea Maribor, Maribor, Slovenia; 5 Faculty of Medicine, Schulich School of Medicine & Dentistry, Western University, London, ON Canada; 6 Institute for Earth and Space Exploration, Western University, London, ON Canada; 7 King’s College London, Centre of Human & Applied Physiological Sciences, London, UK; 8 Space Medicine Team, HRE-OM, European Astronaut Centre, European Space Agency, Cologne, Germany; 9 KBRwyle Laboratories GmbH, Cologne, Germany; 10 Space Biomedicine Systematic Review Methods, Wylam, UK

**Keywords:** Preventive medicine, Physiology

## Abstract

A systematic review of literature was conducted to evaluate the effectiveness of passive countermeasures in ameliorating the cardiopulmonary and musculoskeletal effects of gravitational unloading on humans during spaceflight. This systematic review is the third of a series being conducted by the European Space Agency to evaluate the effectiveness of countermeasures to physiologic deconditioning during spaceflight. With future long-duration space missions on the horizon, it is critical to understand the effectiveness of existing countermeasures to promote astronaut health and improve the probability of future mission success. An updated search for studies examining passive countermeasures was conducted in 2021 to supplement results from a broader search conducted in 2017 for all countermeasures. Ground-based analogue and spaceflight studies were included in the search. A total of 647 articles were screened following removal of duplicates, of which 16 were included in this review. Data extraction and analysis, quality assessment of studies, and transferability of reviewed studies to actual spaceflight based on their bed-rest protocol were conducted using dedicated tools created by the Aerospace Medicine Systematic Review Group. Of the 180 examined outcomes across the reviewed studies, only 20 were shown to have a significant positive effect in favour of the intervention group. Lower body negative pressure was seen to significantly maintain orthostatic tolerance (OT) closer to baseline as comparted to control groups. It also was seen to have mixed efficacy with regards to maintaining resting heart rate close to pre-bed rest values. Whole body vibration significantly maintained many balance-related outcome measures close to pre-bed rest values as compared to control. Skin surface cooling and centrifugation both showed efficacy in maintaining OT. Centrifugation also was seen to have mixed efficacy with regards to maintaining VO2max close to pre-bed rest values. Overall, standalone passive countermeasures showed no significant effect in maintaining 159 unique outcome measures close to their pre-bed rest values as compared to control groups. Risk of bias was rated high or unclear in all studies due to poorly detailed methodologies, poor control of confounding variables, and other sources of bias (i.e. inequitable recruitment of participants leading to a higher male:female ratios). The bed-rest transferability (BR) score varied from 2–7, with a median score of 5. Generally, most studies had good BR transferability but underreported on factors such as control of sunlight or radiation exposure, diet, level of exercise and sleep-cycles. We conclude that: (1) Lack of standardisation of outcome measurement and methodologies has led to large heterogeneity amongst studies; (2) Scarcity of literature and high risk of bias amongst existing studies limits the statistical power of results; and (3) Passive countermeasures have little or no efficacy as standalone measures against cardiopulmonary and musculoskeletal deconditioning induced by spaceflight related to physiologic deterioration due to gravity un-loading.

## Introduction

The human body undergoes numerous adaptations during spaceflight, many of which are sustained following return to Earth^1^. Without gravitational force providing axial head-to-toe loading, astronauts experience thoraco-cephalic fluid redistribution, autonomic nervous system dysregulation, muscle atrophy, and bone demineralisation^1^. Persistent adaptations to spaceflight last for inter-individually variable periods following return to Earth. Some of these adaptations include: orthostatic intolerance^2^, changes in resting heart rate and heart rate variability^3^, exercise intolerance^4^, decreases in postural stability, and changes in gait^5^. Spaceflight induced physiological deconditioning puts astronaut health at risk as well as potentially affecting their performance in completing mission-related tasks^1,6^. Understanding spaceflight deconditioning and its mitigation is of the utmost importance with the onset of exploration-class missions where astronauts will be exposed to longer periods of microgravity followed by variable gravitational environments without access to large medical care and support facilities or resources. Upon landing on a planetary surface, stringent mission timelines, limited access to rehabilitation exercise equipment as well as unknown responses to rehabilitation in hypogravity environments may limit mid-mission rehabilitation efforts. Autonomous functioning of deconditioned astronauts following prolonged hypogravity exposure could threaten both life and mission. Dangerous physiologic effects following spaceflight such as presyncope, perhaps from impaired cerebral blood flow autoregulation^7,8^, could lead to disastrous consequences following transit to the lunar surface. Moreover, it is not known how the human body will react to being exposed to hypogravity after prolonged exposure times to microgravity as foreseen in the current ARTEMIS and Lunar Gateway mission profiles. During the Apollo era, astronauts had only ~3 days of microgravity exposure before they landed on the lunar surface, yet some fell, stumbled, and had other mishaps during their lunar surface EVAs. Current Artemis missions foresee much longer exposure times to microgravity in lunar orbit and it is not known how this will affect crewmembers' orthostatic tolerance and balance when re-exposed to hypogravity on the moon. The moon itself may serve as an effective spaceflight analogue to further research spaceflight deconditioning and can be used as testbed for countermeasures in preparation for further missions to Mars and beyond^9^.

Various countermeasures have been developed and employed by space agencies in order to reduce the risk to astronaut health and increase mission success^1^. Space agencies employ exercise programmes to counteract some of the cardiopulmonary and musculoskeletal effects of microgravity during missions to the International Space Station (ISS)^10,11^. However, exercise as a countermeasure is constrained by mechanical failures, as well as technical, mass and space limitations on space vessels^11^. Pre-establishing in-space and on-surface countermeasure equipment has been postulated as a potential solution to overcome these limitations. However, establishment and use of this equipment comes with its own set of limitations including equipment failure and wear. Added technical complexity to mission planning such as mapping exact trajectories in order to allow for docking of crewmember-containing vessels to orbiting rehabilitation-centres increases the risk of error as well as potentially increasing mission duration. Moreover, evidence suggests that exercise equipment for sustained habitation on the Moon or Mars could be relatively basic to compensate for the “lack” of gravity as simple exercises like rope skipping could be applied to provide a very potent stimulus to maintain musculoskeletal and cardiovascular integrity^12,13^.

Currently, exercise is the primary countermeasure modality used on the ISS. Although it significantly counteracts bone and muscle loss as well as cardiovascular deconditioning in hypogravity—the countermeasure has not been seen to completely ameliorate the adverse cardiopulmonary or musculoskeletal effects of microgravity on the human body^14,15^. A level of acceptable deconditioning will likely need to be established for future exploration-class missions such as the NASA Artemis program^16^. Optimally, this level would allow for safe and efficient completion of mission tasks whilst using the least number of resources and time necessary to achieve. Increased effort and resources have been allocated towards developing effective countermeasures to micro-gravity induced deconditioning for LDSMs on the horizon. Passive countermeasures may have a greater probability of ensuring astronaut health and supporting mission success by maintaining levels of acceptable deconditioning during exploration-class missions which exercise alone may not be able to accomplish.

In this systematic review, passive countermeasures are defined as those wherein the individual using the countermeasure does not need to exert effort for its intended use. Nutritional countermeasures are excluded from this definition as those are examined in a separate dedicated systematic review^17^. To our knowledge, no previous review exists which has evaluated the effectiveness of passive countermeasures as stand-alone means of ameliorating physiologic deconditioning in microgravity. Passive countermeasures are generally used in concert with other types of countermeasures. However, the use of multiple types of countermeasures makes it difficult to discern which countermeasure is affecting measured outcomes and to what efficacy. In order to best establish passive countermeasure effectiveness, they must be examined as standalone measures.

Thus, the aim of this review is to examine the effectiveness of standalone passive countermeasures in preventing cardiopulmonary and musculoskeletal deconditioning in humans due to gravitational unloading in both spaceflight and ground-based analogue studies.

## Results

### Results by passive countermeasure

Although many passive countermeasures have been developed over the last few decades, this review identified five passive countermeasures which have been tested in space analogue studies and were of sufficient quality to be included. Briefly, they include:

### Human centrifugation

Centrifugation involves replacing lost axial loading by gravitational force which humans experience on Earth with induced centripetal force, a countermeasure termed “artificial gravity (AG)”^18^. This force is created through the rotation of an arm at constant velocity around an axis. Space flight size constraints limit short arm centrifuges to a radius of 2 m^19^. In order to create sufficient centrifugal forces to mimic 1-g at the heart using such a short radius, a high rotation rate must be accomplished as the amplitude of centripetal acceleration is directly related to axis arm length^18^. Due to the high spin-rate, there is an increased risk of astronauts experiencing Coriolis cross-coupled illusion which can lead to disorientation and motion sickness^20^. Long-axis centrifuges have the potential to decrease the risk of Coriolis cross-couples illusion as lower spin rates are required to achieve 1 g as compared to their short-axis counterparts. However, many limitations exist which limit these centrifuges’ operational viability. Due to their mass and size, these centrifuges will likely have to be external to the vessel. Additionally, a large external centrifuge will impact navigational trajectory which must be compensated for through expenditure of increased amounts of fuel and added complexity to navigation.

### Lower body negative pressure (LBNP)

LBNP is designed to counteract the thoraco-cephalic fluid shifts experienced in microgravity by simulating gravitational fluid shifts experienced on Earth^21^. The countermeasure also simulates some of the mechanical loading experienced terrestrially and which is lost during spaceflight^22^. Suits that provide LBNP during spaceflight have been used by Russian cosmonauts on the ISS^21^ and a National Aeronautics and Space Administration (NASA)-supported research team has recently developed a mobile LBNP suit for potential use during exploration-class missions^23^.

### Thigh cuffs

Thigh cuffs (aka “bracelets”) apply about 30–40 mmHg of pressure to the thighs in order to reduce venous return to the heart from the lower extremities during spaceflight in order to reduce thoraco-cephalic fluid shift^24^. These devices have been used by Russian cosmonauts for many years, initially to counter the so-called “puffy-face-bird-leg” syndrome^24^.

### Skin surface cooling

Skin surface cooling (SSC) may improve orthostatic tolerance through activation of the sympathetic nervous system, reduction in skin blood flow, increase in mean arterial pressures and preservation of cerebral blood flow velocity. Currently, astronauts wear water-cooled suits only for re-entry or for research purposes^25^.

### Whole body vibration (WBV)

WBV consists of providing 15–45 Hz of sinusoidal vibration to the bottom of the feet through a vibrational platform^26^. The countermeasure works to preserve postural stability by limiting the sensorimotor adaptations which occur in microgravity due to the lack of tactile inputs which normally occur during ambulation on Earth^6^. In those muscles integral for postural stability, these inputs may lead to strength retention as they serve as a surrogate to normal terrestrial muscle loading ^26^.

Notably, there are numerous limitations to consider regarding the operational implementation of WBV for LDSMs. Firstly, WBV may impact microgravity research on crystalline structures through unintended vibrational disruption of these structures^27^. Current Microgravity Vibration Isolation Mounts such as those used on the ISS may be insufficient to attenuate the much larger levels of vibration transmitted through the vessel during WBV. Low amplitude vibration also increases the number of corrective saccadic eye movements needed to visually track a point stimulus^28^. Increasing vibrational exposure of crewmembers could therefore impact vision and coordination during LDSMs. Lastly, the energy dissipated through vibration could impact and damage electronics, further elucidating the importance of isolating vibrational instrumentation.

### Characteristics of reviewed studies

Of the 16 included studies, 9 were randomised control trials (RCTs)^19,25,26,29–34^ whereas 7 were control trials (CTs)^35–41^. All included studies were ground-based analogue studies utilising bed rest (BR) to simulate microgravity-induced physiologic deconditioning. BR period durations of the included studies varied from 7 days to 90 days, with the median being 21 days. A total of 207 participants were included across all the studies. The median number of participants was 11. Only four of the studies included female participants^25,26,31,33^ with a total of 33 female participants. The mean age of participants was 32.5 years (SD = ± 5.20 years).

Nine broad outcome measures reported in the studies were consistent with those included in our search strategy (Table 1). Seven of the studies were conducted in campaigns which included the same participants in countermeasure and control groups^19,32–36,39^. Varying periods of time separated control and countermeasure campaigns in these studies. The remainder of the studies used different participants for control and countermeasure groups. Eleven of the included studies reported cardiopulmonary outcome measures^25,29,30,32–36,38,39,41^, 4 included musculoskeletal performance outcome measures^19,26,33,37^, and two studies included skeletal anthropometric outcome measures^31,40^. A total of five different passive countermeasures were tested across all 16 included studies: centrifugation, LBNP, thigh cuffs, SSC, and WBV.

**Table 1:** Risk bias assessment of included studies

		**Judgement of risk of bias using the Cochrane risk of bias tool**							
**Study type**	**Study**	**Random sequence generation**	**Allocation concealment**	**Blinding of participants and personnel**	**Blinding of outcome assessment**	**Incomplete outcome data**	**Selective reporting**	**Other sources of bias**	**Overall risk of bias**
RCT	^34^	?	?	?	?	−	?	+	?
	^25^	?	?	?	?	−	−	+	?
	^33^	?	?	?	?	−	−	?	?
	^32^	?	?	?	?	+	−	+	+
	^26^	+	+	+	?	+	−	−	+
	^31^	?	?	?	?	−	−	+	?
	^19^	?	?	?	?	+	?	+	+
	^30^	?	?	?	?	−	−	+	?
	^29^	?	?	?	?	−	−	+	?
CT		**Judgement of risk of bias using RoBANS**							
	**Study**	**Selection of participants**	**Confounding variables**	**Intervention measurement**	**Blinding of outcome assessment**	**Incomplete outcome data**	**Selective outcome reporting**	**Overall risk of bias**	
	^35^	+	?	?	?	−	−	?	
	^36^	+	?	−	?	−	−	?	
	^37^	+	?	−	?	−	−	?	
	^38^	+	?	?	?	−	−	?	
	^39^	+	?	−	?	+	−	+	
	^40^	+	−	−	?	−	−	?	
	^41^	+	+	−	?	−	−	+	

+ indicates high risk of bias; − indicates low risk of bias; ? indicates unclear risk of bias.

### Quality assessment of included studies

The results of the risk bias assessment are shown in Table 2. Both assessment tools showed an unclear or high risk of bias in all studies.

**Table 2:** AMSRG bed rest transferability

Study	Bed-rest transferability criteria								Overall transferability score
	Was 6° head-down tilt utilised?	Was diet controlled?	Were sleep-wake cycles controlled?	Were BR phases standardised?	Was horizontal positioning uninterrupted throughout the BR period?	Was sunlight exposure controlled?	Were measurements scheduled to take place at the same time each day?	Was the duration of the BR period stated? (Duration in days)	
^35^	?	?	?	Y	?	?	Y	Y (7)	3
^36^	?	?	?	Y	?	?	?	Y (30)	2
^34^	Y	?	?	Y	N	?	Y	Y (7)	4
^37^	Y	?	?	Y	Y	?	Y	Y (30)	5
^38^	Y	?	?	Y	?	?	?	Y (30)	3
^25^	Y	?	?	Y	Y	?	Y	Y (28)	5
^33^	Y	Y	?	N	Y	?	?	Y (60)	4
^32^	Y	?	?	Y	?	?	?	Y (5)	3
^39^	Y	Y	?	Y	Y	?	Y	Y (7)	6
^26^	Y	?	Y	Y	Y	?	Y	Y (90)	6
^31^	Y	Y	?	Y	N	?	?	Y (90)	4
^19^	Y	Y	Y	Y	Y	?	Y	Y (5)	7
^40^	Y	Y	Y	Y	Y	?	Y	Y (21)	7
^41^	Y	Y	Y	Y	Y	?	Y	Y (21)	7
^30^	Y	Y	?	Y	N	?	Y	Y (21)	5
^29^	Y	Y	?	Y	N	?	Y	Y (21)	5

Y indicated yes, the study met this transferability criterion; N indicates no, the study did not meet this transferability criterion; ? indicates that it is unclear if the study did or did not meet this transferability criterion.

### Bed rest transferability

BR transferability results are summarised in Table 2.

The average BR transferability score was 4.75 with a SD of 1.57. Overall, BR transferability for the included studies varied greatly with a median score of 5. Mostly, a lack of information included within a study’s methodologies contributed to a low score.

### Main outcome parameters

#### Cardiopulmonary outcome measures

**Fig. 1:**
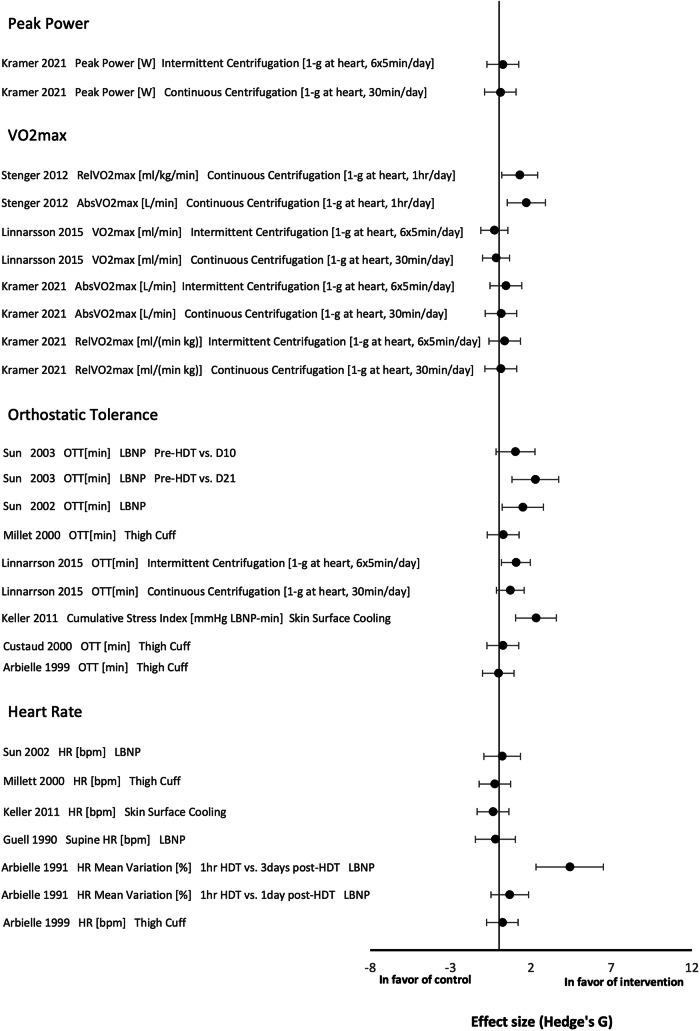
Effect size plot for cardiopulmonary outcome measures. Effect size plot of cardiopulmonary outcomes categorised into “VO2max”, “Orthostatic Tolerance”, and “Heart Rate”. Mean differences between pre and post bed rest values between control and intervention groups were used to calculate effect sizes. Effect sizes were calculated with Hedges’ *G* and bias corrected for sample size with a confidence interval of 95%. A positive value on the *x*-axis demonstrates positive effect of the intervention whereas a negative value demonstrates negative effect of the intervention on the outcome. LBNP is lower-body negative pressure, HDT is head-down tilt, HR is heart rate, OT is orthostatic tolerance, OTT is orthostatic tolerance time (stand test).

Effect sizes comparing control and countermeasure groups for cardiopulmonary outcome measures are displayed in Fig. 1.

#### Peak power

Neither intermittent nor continuous centrifugation were found to have any significant effect on peak power as an outcome measure^33^.

#### VO2Max

Continuous centrifugation was found to have a positive effect on relative and absolute VO2max in one study^41^. However, data from two other studies showed no significant positive or negative effect of centrifugation on VO2max^32,33^.

#### OT

Intermittent centrifugation was found to have a significant positive effect on OT, however continuous centrifugation was not^32^. LBNP data showed that the countermeasure had a significant positive effect when orthostatic tolerance times (OTTs) were compared pre-HDT and after 21 days BR of but not after 10 days of BR^29,30^. Thigh cuffs were not seen to have a significant effect on OTTs^34,35,39^. Skin surface cooling was seen to have a significant positive effect on Cumulative Stress Index, an outcome measure of OT^25^.

#### Heart rate

LBNP was found to have a significant positive effect on HR mean variation three days post-HDT^36^. However, the countermeasure did not have a significant effect on HR one day post-HDT^36^ or in other reviewed studies^30,38^. Thigh cuffs^35,39^ and skin surface cooling^25^ were not found to have significant effects on HR either.

Effect size plots for musculoskeletal performance and skeletal anthropometric outcome measures are available as Supplementary Figs. 1 and 2, respectively, as they are too large for inclusion in the main text. Table 3 summaries the results from all the effect size plots.

**Table 3:** Passive countermeasure effects on studied outcomes derived through effect size analysis

	Outcome measure grouping								
	Cardiopulmonary				MSK performance				Skeletal anthropometric
Passive countermeasure	Peak power	VO2max	OT	HR	Jump	Muscle strength	Gait	Balance	BMD
Centrifugation	=	+/=	+/=	NT	=	=	NT	NT	−/=
Lower-body negative pressure	NT	NT	+/=	+/=	NT	NT	=	NT	NT
Thigh cuffs	NT	NT	=	=	NT	NT	NT	NT	NT
Skin-surface cooling	NT	NT	+	=	NT	NT	NT	NT	NT
Whole body vibration	NT	NT	NT	NT	NT	=	NT	+/=	+/=

+ indicates significant positive effect; = indicates no significant effect; − indicates significant negative effect; NT is not tested within the reviewed studies.

#### Musculoskeletal performance outcome measures

Effect sizes comparing control and countermeasure groups for musculoskeletal performance outcome measures are summarised in Table 3.

#### Jump Performance

Neither continuous nor intermittent centrifugation were found to have significant effects on outcome measures related to jumping^19^.

#### Muscle strength

Neither centrifugation^19,33^ nor WBV were found to have significant effects on muscle strength-related outcome measures^26^.

#### Gait

LBNP was not found to have a significant effect on gait^37^.

#### Balance

Whole Body Vibration was found to have a significant positive effect on many balance-related outcome measures such as anterior-posterior root mean squared velocity with eyes closed at 90 days post-BR; but with no significant effect on others such as anterior-posterior root mean squared velocity with eyes open at 90 days post-BR^26^. Lower Body Negative Pressure was not found to have any significant effect on balance^37^.

#### Skeletal anthropometrics

Effect sizes comparing control and countermeasure groups for skeletal anthropometric outcome measures are summarised in Table 3.

#### Bone Mineral Density

Whole body vibration was found to only have a significant positive effect on ultrasound velocity when baseline data was compared to day 60 data^31^. Ultrasound Velocity (UV) is a component of quantitative ultrasound (QUS), which has been shown to be correlated with BMD in assessing for osteopenia^42^. Otherwise, the countermeasure was not found to have any significant effect on BMD-related outcome measures^31^. Continuous centrifugation was seen to have a significant negative effect on distal polar strength-strain index^40^. Otherwise, continuous centrifugation was not found to have significant effects on any other BMD-related outcome measure^40^.

## Discussion

The results of this systematic review indicate that there are minimal effects of stand-alone passive countermeasures on cardiopulmonary and musculoskeletal outcomes. Additionally, quality of reviewed literature is poor due to high or unclear risks of biases. There is also large heterogeneity between studies, large variations in BR transferability criteria, a lack of standardisation between studies and a paucity of literature resulting in poor statistical power of results.

As with Sandal et al.^17^, this review sought articles examining outcomes in predefined domains within cardiopulmonary and musculoskeletal outcome measures. The absence of standardised outcome measures across the reviewed studies resulted in an inability to conduct a meta-analysis and limited data pooling of results. In fact, many of the reviewed findings are from single studies presenting unique outcome measures. Therefore, a high risk of bias exists with reporting on current findings with recommendations.

The risk of bias within the studies was either high or unclear, mostly attributed to insufficient description of methodologies. None of the RCTs included details of allocation concealment during randomisation^19,25,26,29–34^. However, given the nature of passive countermeasures, blinding of participants may be difficult as participants in the intervention groups undergo daily regimens of obvious countermeasure interventions. Additionally, most of the included RCTs^35–41^ and all of the reviewed CTs^19,29,30,32,34^ included only male participants or had a majority of male participants^25^. The inequitable recruitment of female participants in studies results in a lack of adequate understanding of gender-based differences in the physiologic response to spaceflight^43^. It is highly likely that these differences can have significant impacts on exploration-class mission crewmember health and mission success^43^.

Widely varied BR transferability scores amongst the reviewed studies make direct transfer of review outcomes to actual spaceflight difficult. Although, all the reviewed studies used head-down head tilt BR—various confounding factors may have influenced outcomes to differ from results that would be observed during space flight. For instance adequacy of sleep has been shown to effect athletic performance^44^, muscle strength^45^, and postural stability^46^. Yet, only four studies reported control of the sleep-wake cycles of their participants^19,26,40,41^.

Lower BR transferability scores were largely due to studies not mentioning whether or not certain confounding measures were being controlled. Unfortunately, while perfect simulation of the spaceflight environment is impossible, the validity of direct transfer of the results of microgravity simulation studies to actual spaceflight is likely decreased with low BR transferability score. In this review, none of the outcomes have been pooled and therefore results from reviews with differing BR transferability scores can only be observed independently from one another. Studies examining similar outcome measures but with differing levels of control of confounding variables seem to generally observe similar results with regards to whether or not the examined passive countermeasure had significant positive, negative or no significant effects on outcome measures. It cannot be stated with confidence that the results and conclusions from this review have direct validity for actual spaceflight.

Cardiopulmonary deconditioning during spaceflight presents a major risk to astronaut health^47^. Heart rate decreases in the microgravity environment due to the absence of head-to-foot gravitational force, altering venous return and resulting in structural and functional changes that can persist following return to Earth^47^. Heart rate variability has been used to reflect cardiopulmonary deconditioning in returning astronauts^3^. By reducing venous return to the heart, LBNP simulates some of the gravitational stress experienced by the cardiovascular system on Earth^21^. The current results are mixed in showing either positive^36^ or no significant effect of standalone passive countermeasures on HR^30,38^. It is therefore uncertain if LBNP as a standalone intervention can sufficiently simulate normal physiological stressors on the cardiovascular system to counteract microgravity induced changes to HR. Notably, the positive effect of LBNP on mean HR variation was significant at 3-days post-HDT but not at 1-day post-HDT. One possible explanation for this pertains to the impact of LBNP on the cardiovascular system’s baroreflex feedback loop. Through fluid redistribution and reduction of venous return, LBNP during HDT helps decrease stroke volume, which triggers baroreceptors in the heart and carotid artery to increase heart rate in order to maintain cardiac output (product of heart rate and stroke volume)^48^. This feedback mechanism may not have a significant impact after 1-day post-HDT due to the initial sudden redistribution of fluid (and therefore rapid reduction in heart rate) but may significantly normalise HR to baseline levels by day 3 post-HDT.

Notably, heart rate was also not significantly affected by other passive countermeasures such as thigh cuffs^35,39^ and skin surface cooling^25^.

Decreases in orthostatic tolerance from alterations in autonomic arterial pressure^49^ and postural vasoconstrictor response^2^ have been seen in those returning from spaceflight. LBNP may limit some of the cardiovascular remodelling which occurs in microgravity through provision of “gravity-like” stress on the cardiovascular system^50^. Maintenance of intravascular fluid volume, normalisation of cardiac filling pressures during orthosis, and limitations in cerebral circulatory changes have all been posited as possible explanations as to how the countermeasure can prevent losses in orthostatic tolerance^50^.

In this review, LBNP was seen to improve OTTs during stand-tests following 21 days of BR vs. controls^29,30^. However, it had no significant effect on the same outcome measure following 10 days of BR^29^. Intermittent centrifugation was also seen to have a positive effect on OTT, which the authors found was not related to maintenance of blood volume^32^. Instead, AG is theorised to improve the outcome measure via multiple mechanisms which overall improve sympathetic response to orthostatic loading^32,41^. In the absence of gravitational forces, vasoreactivity of vessels throughout the body are altered secondary to remodelling of vascular smooth muscle^51^. Vasoreactivity increases in cerebral vessels in order to compensate for cerebral hypertension whereas lower limb vessels experience decreases in vasoreactivity^51^. Thus, sympathetic response to orthostatic tolerance is altered due to vascular adaptation. Intermittent AG has been theorised to counteract this vascular adaptation by simulating Earth-like gravitational stress of the cardiovascular system and therefore improving orthostatic tolerance^51^. Supporting this theory, thigh cuffs were not seen to have a significant effect on OTT as they are designed to only maintain plasma volume in the lower limbs^34,35,39^. It is uncertain as to why the positive effects of centrifugation on OTT were not also seen with continuous centrifugation protocols^32^. Skin surface cooling was also found to have a positive effect on OT as indicated by an “Cumulative Stress Index” (CSI)^25^. Cumulative Stress Index was calculated in this study by summing the product of applied negative pressure with the duration of pressure stimulus during LBNP session stages^25^. The authors of this study postulated that this improvement in CSI was due to skin cooling increasing arterial pressure during the study^25^. Other proposed mechanisms include: attenuation in reduction of cerebral blood flow velocity and improvements in sympathetic responses to orthostatic loading^52^.

It is well known that astronauts returning from spaceflight experience reductions in exercise capacity due to physical deconditioning. Aerobic exercise capacity (VO2max) decreases following spaceflight which has been historically postulated as being due to decreases in blood volume^53^. A more recent study examining mechanistic components of oxygen transport demonstrated that decreases in VO2max may instead be due to decreases in O_2_ transport which are affected by cardiovascular system remodelling^54^. Although centrifugation has been seen to perhaps eliminate cardiovascular autonomic changes in microgravity, it has not been shown to improve exercise capacity as a stand-alone countermeasure^55^ which was also seen in another study^32,33^. However, the results from the reviewed studies which showed significant positive effects may have been skewed due to participants performing knee bends and heel raises while using the countermeasure^41^.

Deteriorations in postural stability following spaceflight are thought to be due, in part, to sensorimotor adaptations^6^. These adaptations may occur due to the lack of somatosensory input which humans are accustomed to on Earth^6^. Tactile inputs are important for the preparatory activations which allow for maintenance of postural stability^6^. In astronauts, the absence of these tactile inputs results in alterations in sensitivity to high and low frequency vibrations to their feet, which causes postural instability and alterations in walking^6^. In this way WBV can significantly improve balance-related outcome measures as seen in this review^26^. However, this positive effect was not ubiquitous across all the study conditions. It seems that comparisons of baseline data with BR-day 90 data more often showed no significant effect of the countermeasure. Whereas baseline data vs. BR-day 60 data showed more instances of significant positive effect. This may be due to maximal deterioration in postural stability occurring within 60 days as well as further muscle and endurance deteriorations as the study period progressed^26^. Eyes closed conditions also generally favoured the intervention while eyes open conditions more often showed no significant effect. The authors of the reviewed study postulate that this is due to the importance of visual cues in maintaining balance^26^. Therefore, in the eyes closed conditions, visual cues could not be used by the body to compensate for deteriorations in postural stability caused by deconditioning^26^. Although OT may play a role in balance, it does not appear to be significant as LBNP-induced improvements in OT did not correlate with significant effects on balance nor gait^37^. Therefore, sensorimotor pathways may play a larger role preserving postural stability as opposed to fluid-balance and cardiovascular remodelling. Likely, the sensorimotor adaptation which occurs in microgravity cannot be compensated for with improvements in other systems through LBNP^6^.

Significant muscle atrophy is seen in even short duration space flight^56^. Weight-bearing muscles and those used for gait such as the quadriceps femoris, hamstrings, sartorius, gracilis and triceps surae have been seen to significantly decrease in volume following spaceflight, most likely due to atrophy from the absence of axial loading^57^. The positive benefits previously seen using WBV on plantar flexor and dorsiflexor muscle strength^58^ were not seen in this review^26^. Centrifugation also seems to not provide sufficient loading to preserve muscle strength^19,33^ nor preserve jump-related outcome measures^19^. In the absence of gait and continuous loading by gravity, lower limb muscles will undergo substantial atrophy as loading cannot be sufficiently mimicked with short sessions of centrifugation nor loading through WBV.

Similar to muscle atrophy caused by unloading, disuse osteopenia presents a significant threat to astronaut health during exploration-class missions^31,40^. WBV has been seen in some studies to increase BMD and bone anabolism and has even been theorised as a treatment for osteoporosis^59^. In this review WBV was only seen to significantly positively effect UV^31^. However, the absence of effects on other components of QUS or BMD in the reviewed study^31^ make it unlikely that there was true significant positive benefit of WBV on bone density. As with other discussed outcome measures, it seems that WBV may not provide enough mechanical stimulation to simulate axial-loading on Earth and therefore cannot significantly alter micro-gravity induced bone catabolism. This is also true for the absence of a positive effect of centrifugation as seen in this review^40^. The reasoning for the significant negative effect of centrifugation on polar strength-strain index (SSI P—a surrogate marker of bone strength) of the distal tibia is uncertain.

This review aimed to examine the effectiveness of stand-alone passive countermeasures on cardiopulmonary and musculoskeletal deconditioning in humans due to gravitational unloading. This study had three main findings: (1) a lack of standardisation of outcome measurement and methodologies has led to a large amount of heterogeneity amongst studies; (2) overall, standalone passive countermeasures seem to have little significant effect on cardiopulmonary and musculoskeletal outcomes; and (3) scarcity of literature and high risk of bias amongst existing studies limits the statistical power of results.

The results of this review are pertinent to future deep space and planetary surface explorations, particularly as the space industry transitions from Low-Earth Orbit (LEO) missions to exploration-class missions. NASA’s Artemis program is an example of such an endeavour^60^. As early as 2024, humans will be sent to the moon for the first time in over five decades. The Artemis program is a herald to the paradigm shift occurring within the field of aerospace medicine. Currently, the goals of both passive and active countermeasures tested and utilised on the ISS are largely to maintain baseline human physiology during LEO missions.

Most spaceflight countermeasure studies examine the degree to which human physiology can be maintained close to baseline—that is to what degree measured physiologic parameters can be maintained in-flight and post-flight as compared to subjects’ normal physiology on Earth. As humanity transitions to longer distance and duration space travel, there must be some level of acceptance of deconditioning as it will prove difficult and perhaps operationally unnecessary to endeavour to completely maintain crewmembers at their baseline physiology. Classically used outcome measures for LEO spaceflight studies such as VO2max, BMD and muscle strength will be of less importance during planned Artemis missions as crewmember deconditioning of these parameters is unlikely to significantly impact mission success or threaten crewmember life. Instead, maintaining crewmember operational functionality with respect to orthostatic tolerance, balance-related outcomes, spatial orientation, and postural stability may be of increased importance as they perform extravehicular activities (EVAs) on the lunar surface.

The most critical point in time for crewmember health during the Artemis lunar surface missions will be during the transition from microgravity to lunar hypogravity. Many knowledge gaps exist with regards to how the physiologic toll of spaceflight coupled with the transition to a hypogravity environment will influence the ability of crewmembers to complete mission tasks. NASA has identified a significant research gap regarding the effect of lunar hypogravity on orthostatic intolerance^61^. The many effects of orthostatic intolerance such as headaches, vision changes, presyncope and syncope can prove extremely dangerous to crewmembers particularly during lunar EVAs. Although there may be some suggestion of protective benefits of lunar hypogravity on crewmember exposure to microgravity during transit; existing simulation studies are limited in number, quality, and transferability to actual spaceflight. Current exercise countermeasures during LEO missions and volume resuscitation upon landing have limited orthostatic intolerance in returning crewmembers^62^. Yet the same level of use of these countermeasures may be difficult or impossible during Artemis missions given resource constraints. Moreover, the extended duration of exposure to microgravity during Artemis missions, the much smaller gravitational loading present on the moon as compared to Earth, and the absence of availability of post-flight rehabilitation measures are some of the factors which may be barriers to recovery of OT following transit to the lunar surface as compared to return to Earth from LEO missions. During the Apollo program, astronauts were placed on the lunar surface after only 3 days of transit through space. In comparison, Artemis missions will likely require much longer durations of exposure to microgravity. Treadmills and/or advance resistive exercise equipment currently available for LEO missions may not be available during these missions. Operational space may be available on the Lunar Gateway however, overall, current limitations on the accessibility of exercise further highlight the potential importance of passive countermeasure utilisation.

Although limited in statistical power, the results of this review indicate that LBNP and skin-surface cooling demonstrated a significant positive effect on OT as standalone measures. Returning crewmembers have also been found to have experienced transient declines in postural stability and sensorimotor control which has led to post-flight disturbances in coordination, balance, and mobility^63^. These deteriorations are thought to stem from a lack of somatosensory stimulation during spaceflight as it leads to transient functional vestibular deficiency^64^. As mentioned in this review, mechanical stimulation through vibrational plates has been suggested and studied as a possible countermeasure to this deterioration. Additionally, counterpressure garments may have potential benefits for orthosis following lunar and Mars surface landing. Previously, counterpressure garments have been seen to have initial benefits on orthosis for crewmembers following return from LEO. These studies results could not be included in the data analysed in this review, however, due to potential confounding of outcomes during spaceflight secondary to the use of other countermeasures over the studies’ durations (i.e. exercise)^65–67^.

Spaceflight-Associated Neuro-Ocular Syndrome (SANS) includes a spectrum of findings and symptoms which have been seen to affect astronauts during long-duration space missions^21,68^. Optic disc oedema is the cardinal sign of SANS with globe flattening, cotton wool spots, choroidal and retinal folds, and hyperopic refractive error also often observed^21,68^. There is a potential of SANS to impact crewmember ability to safely and efficiently complete mission tasks with the main reported symptom being decreased near vision acuity^21^. These symptoms have been reported in individuals in as little as 3 weeks following microgravity exposure^69^. Although disease aetiology is poorly understood, a large component of SANS is thought to originate from increased intracranial pressure as a result of microgravity-induced cephalad fluid shift^21,68^. LBNP is being explored as a potential modality to limit the occurrence/severity of SANS due to its limiting of this fluid shift^21^. However, numerous unknowns must first be addressed prior to the use of LBNP for ameliorating the occurrence of SANS, as discussed in Harris et al.^21^. Some of these unknows include understanding the process of physiologic adaptation leading to SANS, how effective LBNP is at ameliorating SANS, how long the modality would need to be used for^21^, and whether there is an acceptable severity of SANS which needs to be accepted as a consequence of LDSM prolonged microgravity exposure. There is a need for further research to explore and answer these questions particularly considering how SANS may impact LDSMs on the horizon.

Although this study shows the potential positive effects of LBNP, skin-surface cooling and WBV on operationally-relevant outcome measures, these results were limited by the quality, quantity and transferability of extracted data. Therefore, this review serves to highlight these countermeasures as a possible focus for future research and demonstrates the potential of passive countermeasures to aid in the success of future space missions. There is a need for future studies to further examine the utility of passive countermeasures and their role for future spaceflight. Additionally, numerous passive countermeasures have not been adequately examined in CTs and RCTs such as compression clothing, electromagnetic stimulation, and specialised garments. As humanity moves towards deep space and planetary surface exploration, it is imperative that these knowledge gaps are filled to ensure mission success and protect crew health.

## Methods

The European Space Agency’s (ESA) Space Medicine Team (SMT) is completing a systematic review series evaluating evidence regarding passive, active and nutritional countermeasures to microgravity-induced pathologies. The literature being examined is a mix of inflight studies as well as ground-based analogues. This review is the third in the series and the first to examine stand-alone passive countermeasures^17,70^. Data extraction, effect size calculation and quality assessment of literature were done using tools created by Aerospace Medicine SR Group (ASMRG)^71^.

### Search strategy

An initial search was conducted and presented in 2017 by Fiebig et al.^17^ using several keywords and groupings (Table 4). These keywords were combined with Boolean logic as shown in Table 4. The main and specific categories as well as the overall search strategy were defined by the European Astronaut Centre space physiology and space medicine operations experts. Articles in English from the following databases were included: Pubmed, Web of Science, Embase, Institute of Electrical and Electronics Engineers (IEEE) database, the ESA’s “Erasmus Experiment Archive”, the NASA “Life Science Data Archive” and “Technical Reports Server” and the German Aerospace Centre’s (DLR) database.

**Table 4:** ESA Space Medicine Team search strategy 2017 taken from Sandal et al. ^17^

Main category	Specific category		Keywords in Boolean search format	Search number	Search mask
Microgravity	Synonyms		“space analogue” OR “ground-based analogue” OR “terrestrial analogue” OR “space flight” OR space-flight OR spaceflight OR “Space mission” OR “space station” OR “micro gravity” OR micro-gravity OR microgravity OR spaceflight OR weightless* OR “orbital flight” OR “zero gravity” OR “space shuttle”	1	Abstract/ Title
	Methods & simulations		“bed rest” OR bed-rest OR “dry immersion” OR dry-immersion	2	Abstract/ Title
			#1 AND #2	3	
	Population of interest		Astronaut* OR astronaut [Mesh] OR cosmonaut* OR taikonaut*	4	Abstract/ Title
			#1 OR #3 OR #4	5	
Countermeasures	Active countermeasures		Countermeasure* OR exercis* OR exercise [Mesh] OR sport* OR “physical activity” OR “physically active”	6	All Fields
	Passive countermeasures		Centrifug* OR suit* OR “lower body negative pressure” OR LBNP or “fluid loading” OR garment OR stimulation OR “artificial gravity” OR “axial loading” OR electromyostimulation OR “electrical muscle stimulation” OR EMS OR “neuromuscular electrical stimulation” OR NMES OR “whole body vibration” OR WBV	7	All Fields
	Nutritional countermeasures		Diet, food, and nutrition [Mesh] OR nutrition* OR diet* OR food* OR supplement* OR protein* OR salt OR saline OR bi-phosphonate OR phosphonate OR nucleotide* OR vitamin*	8	All Fields
			#6 OR #7 OR #8	9	
Operationally relevant outcome parameters	Cardiopulmonary & -vascular	Physical performance	“endurance” OR Vo2 OR Vo2max OR Vo2peak OR “maximal oxygen uptake” OR “peak oxygen uptake” OR “resting heart rate” OR “peak power” OR “maximal work load” OR “orthostatic tolerance” OR “orthostatic intolerance” OR “time until presyncope” OR “exercise tolerance” OR “central fatigue” OR “threshold” OR “onset of blood lactate accumulation” OR “OBLA”	10	All Fields
	Musculoskeletal / Biomechanical	Physical performance	“muscle strength” OR “muscular strength” OR “muscle function” OR “muscular function” OR “muscle power” OR “muscular power” OR “muscle force” OR “muscular force” OR fatigability OR “fatigue resistance” OR “peripheral fatigue” OR “joint moment” OR “joint moments” OR “postural stability” OR posture OR “postural control” OR balance OR sway OR motion OR locomotion OR gait OR walk* OR run* OR jump* OR hop* OR “movement quality” OR “movement pattern” OR “motion pattern” OR coordination OR “motor control” OR “core stability” OR “core strength” OR “trunk stability” OR “trunk strength” OR “lumbopelvic stability” OR “lumbopelvic stability” OR “lumbopelvic control” OR “lumbopelvic control”	11	All Fields
		Anthropometrics	Anthropometr* OR “skeletal strength” OR “bone mineral density” OR “bone density” OR “bone mineral content” OR flexib* OR “range of movement” OR “range of motion”	12	All Fields
			#10 OR #11 OR #12	13	
			#5 AND #9 AND #13	14	
			Apply human filter		

In May of 2021, an updated search was performed for databases which had produced the majority of the relevant articles during the 2017 search: Pubmed, Web of Science, and Embase. For this update, the search strategy was modified to only include articles examining passive countermeasures. Keywords used to search for active and nutritional countermeasure studies were not included (search numbers 6, 8 and 9 in Table 4). Exclusion of these keywords required adjustment in Boolean operators (#9 was replaced with #7 in search #14 within Table 4).

**Fig. 2:**
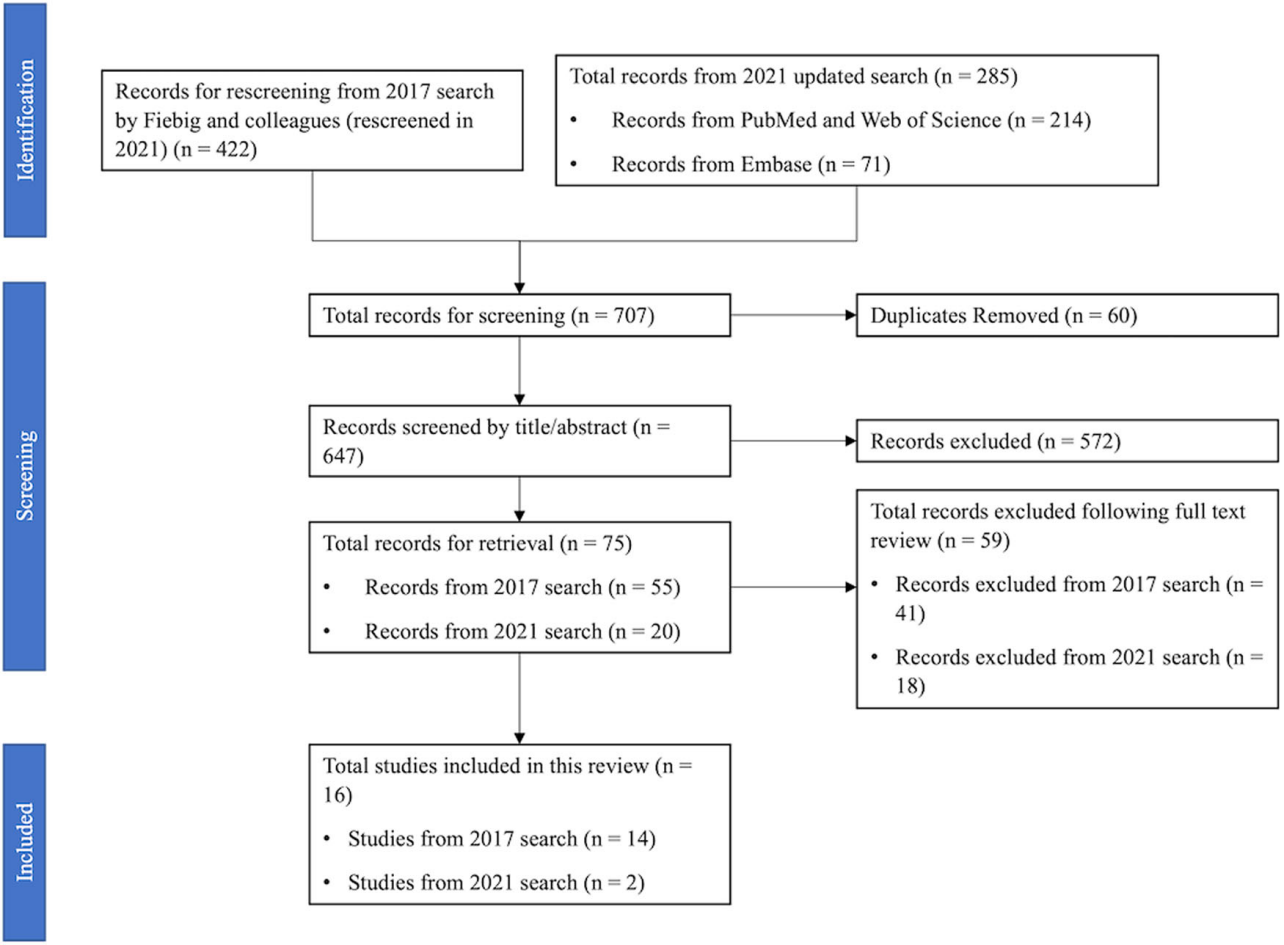
PRISMA. Diagram mapping the number of records identified for inclusion in this review following the 2021 updated search.

In 2017, Fiebig and colleagues screened the titles and abstracts of *n* = 2695 unique records which the initial search yielded^17^. *n* = 433 unique records pertaining to passive countermeasures were identified to meet inclusion criteria based on title/abstract screening. These records were rescreened in 2021. The updated 2021 search yielded *n* = 286 results. All records (*n* = 647 following duplicate removal) were screened using established PICOS as defined in Sandal et al.^17^: “*Population*: Healthy humans; *Interventions:* Space flights and ground-based space flight analogues with a duration of ≥5 days with a stand-alone passive countermeasure; *Control*: Space flights and ground-based space flight analogues with a duration of ≥5 days without any form of countermeasure; *Outcomes*: Studies must contain cardiopulmonary/vascular and/or musculoskeletal/biomechanical outcome measures; and, *Study designs*: Randomized control trials (RCT) and controlled clinical trial (CT)”. The PRISMA flow diagram for the updated search is shown below (Fig. 2).

### Data collection and analysis

Double blinded screening of articles was accomplished through use of the online software tool Rayyan^72^. Two screeners independently decided if articles met established inclusion criteria. A third screener resolved any disagreements. As detailed above, in 2017, two independent reviewers screened for ground-based analogue and spaceflight studies that examined countermeasures grouped into active, passive, nutritional and mixed countermeasures^17^. In 2021, an updated search was completed through the Pubmed, Web of Science and Embase databases. Following this search, two other independent reviewers did the abstract and title screening for passive countermeasure studies using records that passed title/abstract screening in 2017 in addition to records found through the updated search (Fig. 2). Full text versions of the resulting articles following title and abstract screening were obtained and screened further using the PICOS inclusion criteria. A third independent reviewer resolved any disagreements amongst the other two with regards to inclusion or exclusion as required. It should be noted that although spaceflight studies were screened, none met inclusion criteria due to risk of confounding as study participants concurrently used exercise and/or other countermeasures during study durations. Therefore, it would have been difficult to truly assess the effectiveness of passive countermeasure being studied as standalone measures.

### Data extraction

The ASMRG Data Extraction and Analysis form was used to extract data from the included studies^71^. This form was created by the ASMRG as a tool to standardise data extraction and analysis for systematic reviews in aerospace medicine completed by the group. Means and standard deviations of control vs. countermeasure groups pre- and post- BR protocol were extracted from included studies and entered into the AMSRG form in order to calculate effect sizes.

### Quality assessment

The lead author assessed the risk of bias for each of the included studies using one of two tools depending on whether the included articles were RCTs or CTs. For RCTs, a risk of bias tool curated by the Space Biomedicine SR Methods Group was utilised^17^. For non-randomised CTs, the Risk of Bias Assessment for Non-Randomised Studies (RoBANS) was used to evaluate risk of bias^73^.

### Bed rest transferability

The ASMRG bed-rest (BR) transferability tool allows for ground-based analogue studies using BR to be assessed on their ability to mimic microgravity conditions on the human body as experienced during actual human spaceflight^74^. The tool is used to evaluate each study on 8 different criteria with points awarded if a criterion is met. These criteria were developed from the International Academy of Aeronautics’ (IAA) *Guidelines for Standardization of Bed Rest Studies in the Spaceflight Context*^75^. The more criteria which are met, the higher the score and therefore the greater the transferability of the study to human space flight^74^. Transferability is ranked from 1 (poor) to 8 (excellent). The 8 criteria are as follows: (1) Was 6° head-down tilt utilised?; (2) Was diet controlled?; (3) Were sleep-wake cycles controlled?; (4) Were BR phases standardised?; (5) Was horizontal positioning uninterrupted throughout the BR period?; (6) Was sunlight exposure controlled?; (7) Were measurements scheduled to take place at the same time each day?; and (8) Was the duration of the BR period stated?

### Data analysis

A full meta-analysis could not be performed due to the heterogeneity of the extracted data. Means and standard deviations were extracted from the included studies in order to calculate effect sizes based on mean differences of control vs. countermeasure groups pre- and post- BR. Due to the small sample sizes of the included studies, Hedge’s G method was used for correction of bias wherein the quotient of the difference of means is taken from the pooled standard deviation^17^. As in Sandal et al.^17^, data were presented in effect size plots with 95% confidence interval bars. Outcomes were grouped by the broad groupings reflecting those used in the search strategy: “cardiopulmonary,” “musculoskeletal performance” and “skeletal anthropometric”. Outcomes were then further grouped by specific variables being measured. The cardiopulmonary grouping included: heart rate (HR), orthostatic tolerance (OT), VO2max and peak power. The musculoskeletal performance grouping included: balance, gait, muscle strength and jump performance. Finally, the skeletal anthropometric grouping included bone mineral density (BMD).

### Reporting summary

Further information on research design is available in the Nature Research Reporting Summary linked to this article.

### Supplementary information

Supplementary Information

Reporting Summary

## Data Availability

Data extracted from reviewed articles and used for effect size analysis are available upon request.

## Code availability

The European Astronaut Center’s Space Medicine Systematic Review Group’s data analysis excel sheet used for data analysis is available upon request.
