# Supplementary material for: Systematic review of the effectiveness of standalone passive countermeasures on microgravity-induced physiologic deconditioning
Source: NPJ Microgravity. 2024 Apr 25;10:48. doi: 10.1038/s41526-024-00389-1 (PMC11045828; doi:10.1038/s41526-024-00389-1)
Supplement: Supplementary file 1 — Supplementary Information [file 41526_2024_389_MOESM1_ESM.pdf]

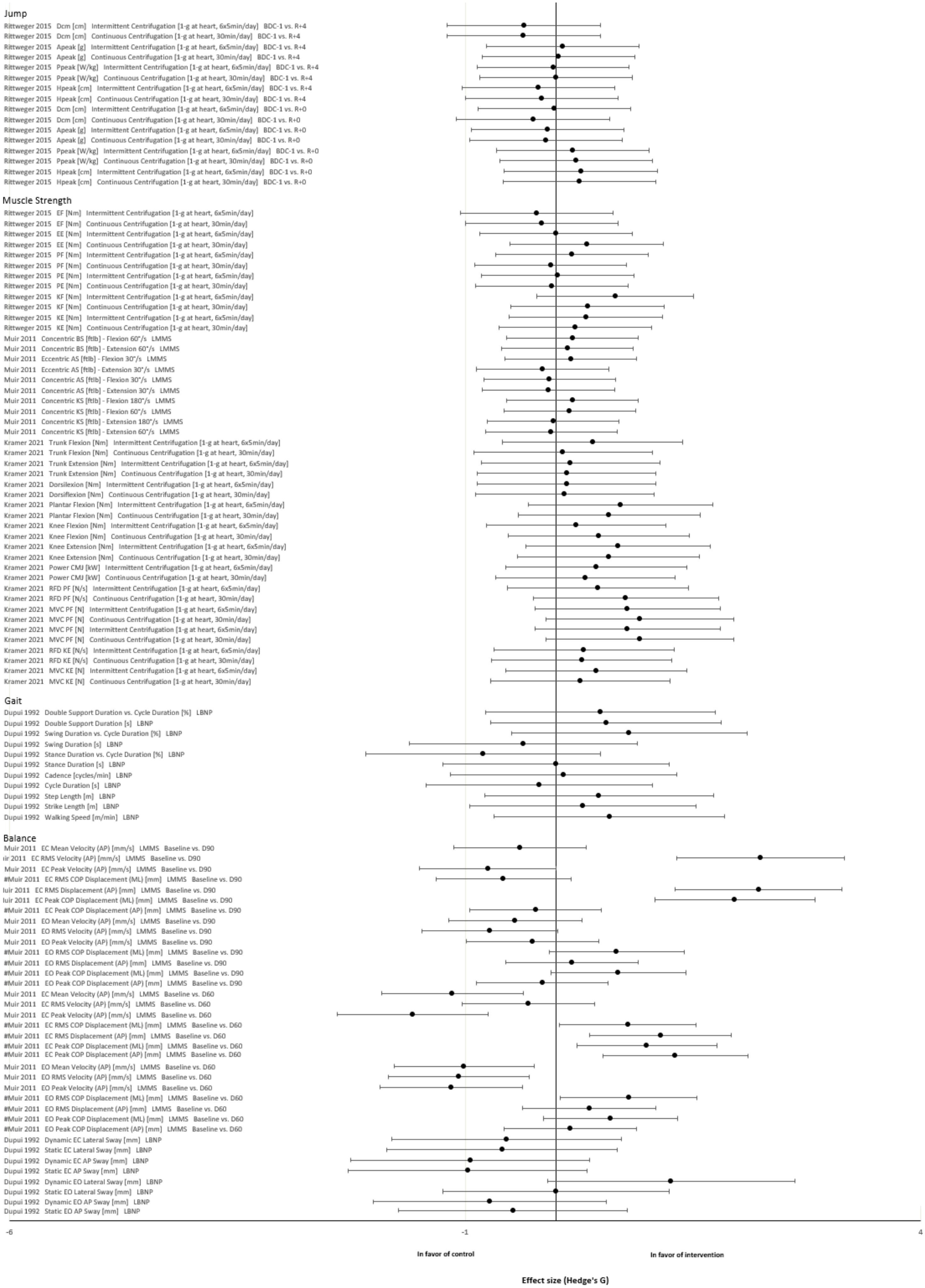
Jump
Rittweger 2015 Dcm [cm] Intermittent Centrifugation [1-g at heart, 6x5min/day] BDC-1 vs. R+4
Rittweger 2015 Dcm [cm] Continuous Centrifugation [1-g at heart, 30min/day] BDC-1 vs. R+4
Rittweger 2015 Apeak [g] Intermittent Centrifugation [1-g at heart, 6x5min/day] BDC-1 vs. R+4
Rittweger 2015 Apeak [g] Continuous Centrifugation [1-g at heart, 30min/day] BDC-1 vs. R+4
Rittweger 2015 Ppeak [W/kg] Intermittent Centrifugation [1-g at heart, 6x5min/day] BDC-1 vs. R+4
Rittweger 2015 Ppeak [W/kg] Continuous Centrifugation [1-g at heart, 30min/day] BDC-1 vs. R+4
Rittweger 2015 Hpeak [cm] Intermittent Centrifugation [1-g at heart, 6x5min/day] BDC-1 vs. R+4
Rittweger 2015 Hpeak [cm] Continuous Centrifugation [1-g at heart, 30min/day] BDC-1 vs. R+4
Rittweger 2015 Dcm [cm] Intermittent Centrifugation [1-g at heart, 6x5min/day] BDC-1 vs. R+0
Rittweger 2015 Dcm [cm] Continuous Centrifugation [1-g at heart, 30min/day] BDC-1 vs. R+0
Rittweger 2015 Apeak [g] Intermittent Centrifugation [1-g at heart, 6x5min/day] BDC-1 vs. R+0
Rittweger 2015 Apeak [g] Continuous Centrifugation [1-g at heart, 30min/day] BDC-1 vs. R+0
Rittweger 2015 Ppeak [W/kg] Intermittent Centrifugation [1-g at heart, 6x5min/day] BDC-1 vs. R+0
Rittweger 2015 Ppeak [W/kg] Continuous Centrifugation [1-g at heart, 30min/day] BDC-1 vs. R+0
Rittweger 2015 Hpeak [cm] Intermittent Centrifugation [1-g at heart, 6x5min/day] BDC-1 vs. R+0
Rittweger 2015 Hpeak [cm] Continuous Centrifugation [1-g at heart, 30min/day] BDC-1 vs. R+0
Muscle Strength
Rittweger 2015 EF [Nm] Intermittent Centrifugation [1-g at heart, 6x5min/day]
Rittweger 2015 EF [Nm] Continuous Centrifugation [1-g at heart, 30min/day]
Rittweger 2015 EE [Nm] Intermittent Centrifugation [1-g at heart, 6x5min/day]
Rittweger 2015 EE [Nm] Continuous Centrifugation [1-g at heart, 30min/day]
Rittweger 2015 PF [Nm] Intermittent Centrifugation [1-g at heart, 6x5min/day]
Rittweger 2015 PF [Nm] Continuous Centrifugation [1-g at heart, 30min/day]
Rittweger 2015 PE [Nm] Intermittent Centrifugation [1-g at heart, 6x5min/day]
Rittweger 2015 PE [Nm] Continuous Centrifugation [1-g at heart, 30min/day]
Rittweger 2015 KF [Nm] Intermittent Centrifugation [1-g at heart, 6x5min/day]
Rittweger 2015 KF [Nm] Continuous Centrifugation [1-g at heart, 30min/day]
Rittweger 2015 KE [Nm] Intermittent Centrifugation [1-g at heart, 6x5min/day]
Rittweger 2015 KE [Nm] Continuous Centrifugation [1-g at heart, 30min/day]
Muir 2011 Concentric BS [ftlb] - Flexion 60°/s LMMS
Muir 2011 Concentric BS [ftlb] - Extension 60°/s LMMS
Muir 2011 Eccentric AS [ftlb] - Flexion 30°/s LMMS
Muir 2011 Eccentric AS [ftlb] - Extension 30°/s LMMS
Muir 2011 Concentric AS [ftlb] - Flexion 30°/s LMMS
Muir 2011 Concentric AS [ftlb] - Extension 30°/s LMMS
Muir 2011 Concentric KS [ftlb] - Flexion 180°/s LMMS
Muir 2011 Concentric KS [ftlb] - Flexion 60°/s LMMS
Muir 2011 Concentric KS [ftlb] - Extension 180°/s LMMS
Muir 2011 Concentric KS [ftlb] - Extension 60°/s LMMS
Kramer 2021 Trunk Flexion [Nm] Intermittent Centrifugation [1-g at heart, 6x5min/day]
Kramer 2021 Trunk Flexion [Nm] Continuous Centrifugation [1-g at heart, 30min/day]
Kramer 2021 Trunk Extension [Nm] Intermittent Centrifugation [1-g at heart, 6x5min/day]
Kramer 2021 Trunk Extension [Nm] Continuous Centrifugation [1-g at heart, 30min/day]
Kramer 2021 Dorsilexion [Nm] Intermittent Centrifugation [1-g at heart, 6x5min/day]
Kramer 2021 Dorsiflexion [Nm] Continuous Centrifugation [1-g at heart, 30min/day]
Kramer 2021 Plantar Flexion [Nm] Intermittent Centrifugation [1-g at heart, 6x5min/day]
Kramer 2021 Plantar Flexion [Nm] Continuous Centrifugation [1-g at heart, 30min/day]
Kramer 2021 Knee Flexion [Nm] Intermittent Centrifugation [1-g at heart, 6x5min/day]
Kramer 2021 Knee Flexion [Nm] Continuous Centrifugation [1-g at heart, 30min/day]
Kramer 2021 Knee Extension [Nm] Intermittent Centrifugation [1-g at heart, 6x5min/day]
Kramer 2021 Knee Extension [Nm] Continuous Centrifugation [1-g at heart, 30min/day]
Kramer 2021 Power CMJ [kW] Intermittent Centrifugation [1-g at heart, 6x5min/day]
Kramer 2021 Power CMJ [kW] Continuous Centrifugation [1-g at heart, 30min/day]
Kramer 2021 RFD PF [N/s] Intermittent Centrifugation [1-g at heart, 6x5min/day]
Kramer 2021 RFD PF [N/s] Continuous Centrifugation [1-g at heart, 30min/day]
Kramer 2021 MVC PF [N] Intermittent Centrifugation [1-g at heart, 6x5min/day]
Kramer 2021 MVC PF [N] Continuous Centrifugation [1-g at heart, 30min/day]
Kramer 2021 MVC PF [N] Intermittent Centrifugation [1-g at heart, 6x5min/day]
Kramer 2021 MVC PF [N] Continuous Centrifugation [1-g at heart, 30min/day]
Kramer 2021 RFD KE [N/s] Intermittent Centrifugation [1-g at heart, 6x5min/day]
Kramer 2021 RFD KE [N/s] Continuous Centrifugation [1-g at heart, 30min/day]
Kramer 2021 MVC KE [N] Intermittent Centrifugation [1-g at heart, 6x5min/day]
Kramer 2021 MVC KE [N] Continuous Centrifugation [1-g at heart, 30min/day]
Gait
Dupui 1992 Double Support Duration vs. Cycle Duration [%] LBNP
Dupui 1992 Double Support Duration [s] LBNP
Dupui 1992 Swing Duration vs. Cycle Duration [%] LBNP
Dupui 1992 Swing Duration [s] LBNP
Dupui 1992 Stance Duration vs. Cycle Duration [%] LBNP
Dupui 1992 Stance Duration [s] LBNP
Dupui 1992 Cadence [cycles/min] LBNP
Dupui 1992 Cycle Duration [s] LBNP
Dupui 1992 Step Length [m] LBNP
Dupui 1992 Strike Length [m] LBNP
Dupui 1992 Walking Speed [m/min] LBNP
Balance
Muir 2011 EC Mean Velocity (AP) [mm/s] LMMS Baseline vs. D90
iir 2011 EC RMS Velocity (AP) [mm/s] LMMS Baseline vs. D90
Muir 2011 EC Peak Velocity (AP) [mm/s] LMMS Baseline vs. D90
#Muir 2011 EC RMS COP Displacement (ML) [mm] LMMS Baseline vs. D90
luir 2011 EC RMS Displacement (AP) [mm] LMMS Baseline vs. D90
luir 2011 EC Peak COP Displacement (ML) [mm] LMMS Baseline vs. D90
#Muir 2011 EC Peak COP Displacement (AP) [mm] LMMS Baseline vs. D90
Muir 2011 EO Mean Velocity (AP) [mm/s] LMMS Baseline vs. D90
Muir 2011 EO RMS Velocity (AP) [mm/s] LMMS Baseline vs. D90
Muir 2011 EO Peak Velocity (AP) [mm/s] LMMS Baseline vs. D90
#Muir 2011 EO RMS COP Displacement (ML) [mm] LMMS Baseline vs. D90
#Muir 2011 EO RMS Displacement (AP) [mm] LMMS Baseline vs. D90
#Muir 2011 EO Peak COP Displacement (ML) [mm] LMMS Baseline vs. D90
#Muir 2011 EO Peak COP Displacement (AP) [mm] LMMS Baseline vs. D90
Muir 2011 EC Mean Velocity (AP) [mm/s] LMMS Baseline vs. D60
Muir 2011 EC RMS Velocity (AP) [mm/s] LMMS Baseline vs. D60
Muir 2011 EC Peak Velocity (AP) [mm/s] LMMS Baseline vs. D60
#Muir 2011 EC RMS COP Displacement (ML) [mm] LMMS Baseline vs. D60
#Muir 2011 EC RMS Displacement (AP) [mm] LMMS Baseline vs. D60
#Muir 2011 EC Peak COP Displacement (ML) [mm] LMMS Baseline vs. D60
#Muir 2011 EC Peak COP Displacement (AP) [mm] LMMS Baseline vs. D60
Muir 2011 EO Mean Velocity (AP) [mm/s] LMMS Baseline vs. D60
Muir 2011 EO RMS Velocity (AP) [mm/s] LMMS Baseline vs. D60
Muir 2011 EO Peak Velocity (AP) [mm/s] LMMS Baseline vs. D60
#Muir 2011 EO RMS COP Displacement (ML) [mm] LMMS Baseline vs. D60
#Muir 2011 EO RMS Displacement (AP) [mm] LMMS Baseline vs. D60
#Muir 2011 EO Peak COP Displacement (ML) [mm] LMMS Baseline vs. D60
#Muir 2011 EO Peak COP Displacement (AP) [mm] LMMS Baseline vs. D60
Dupui 1992 Dynamic EC Lateral Sway [mm] LBNP
Dupui 1992 Static EC Lateral Sway [mm] LBNP
Dupui 1992 Dynamic EC AP Sway [mm] LBNP
Dupui 1992 Static EC AP Sway [mm] LBNP
Dupui 1992 Dynamic EO Lateral Sway [mm] LBNP
Dupui 1992 Static EO Lateral Sway [mm] LBNP
Dupui 1992 Dynamic EO AP Sway [mm] LBNP
Dupui 1992 Static EO AP Sway [mm] LBNP
-6
-1
4
In favor of control
In favor of intervention
Effect size (Hedge's G)

Supplementary Figure 1: Effect Size Plot for Musculoskeletal Performance Outcome Measures. Effect size plot of musculoskeletal performance outcomes categorized into ‘Jump’, ‘Muscle Strength’, ‘Gait’; and ‘Balance’. Mean differences between pre and post bed rest values between control and intervention groups were used to calculate effect sizes. Effect sizes were calculated with Hedges’ G and bias corrected for sample size with a confidence interval of 95%. A positive value on the x-axis demonstrates positive effect of the intervention whereas a negative value demonstrates negative effect of the intervention on the outcome. Dcm = Countermovement depth; Apeak = Peak acceleration; Ppeak = Peak power specific to body mass; Hpeak = Height peak; BDC = Baseline data collection; R = Recovery; CMJ = Countermovement jump; RFD = Rate of force development; MVC = Maximal voluntary contraction; EF = Elbow flexion; EE = Elbow extension; PF = Plantar flexion; PE = Plantar extension; KE = Knee extension; KF = Knee flexion; COP = Center of pressure; AP = Anteroposterior; ML = Mediolateral; EC = Eyes closed; EO = Eyes open.

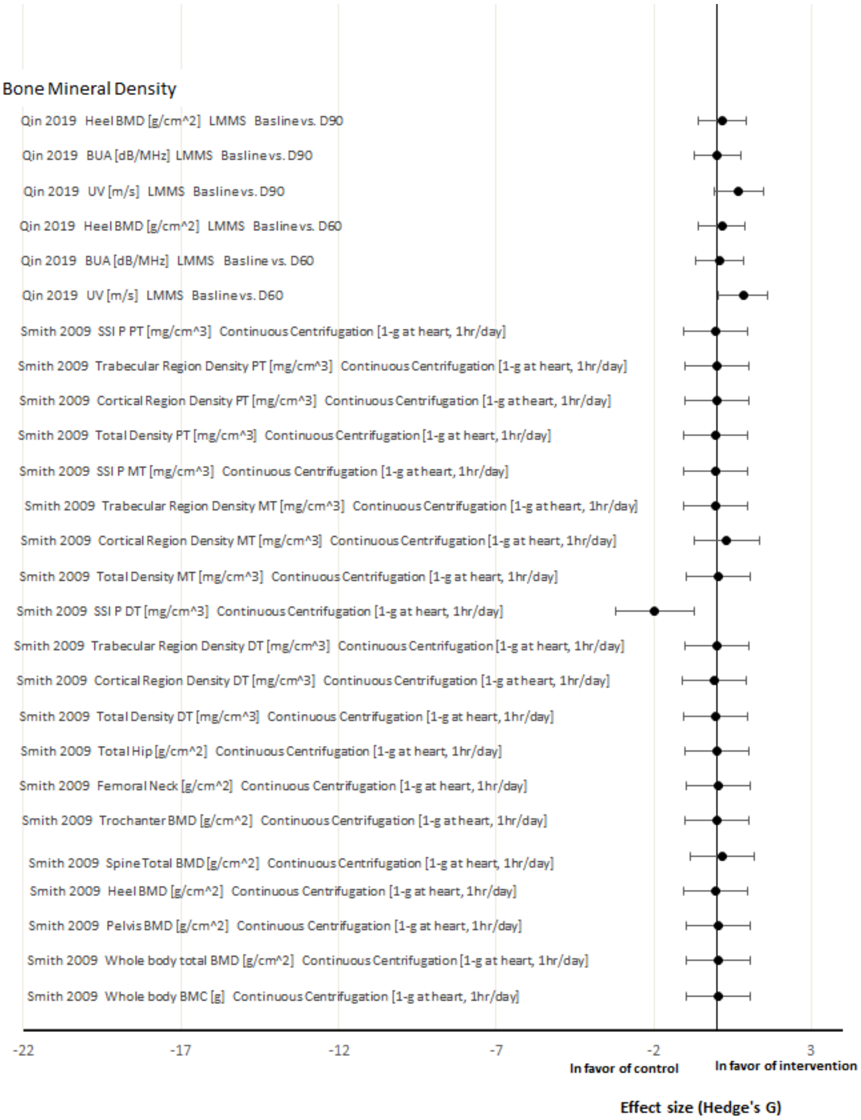

Supplementary Figure 2: Effect Size Plot for Skeletal anthropometric Outcome Measures. Effect size plot of skeletal anthropometric outcomes categorized into 'Bone Mineral Density'. Mean differences between pre and post bed rest values between control and intervention groups were used to calculate effect sizes. Effect sizes were calculated with Hedges' G and bias corrected for sample size with a confidence interval of 95%. A positive value on the x-axis demonstrates positive effect of the intervention whereas a negative value demonstrates negative effect of the intervention on the outcome. BUA = Broadband ultrasound attenuation; UV = Ultrasound velocity; SSI P = Polar strength-strain index; PT = Proximal tibia; DT = Distal tibia; MT = Medial tibia
